# An Exploratory Retrospective Comparison of Crisugabalin and Pregabalin Among 12‐Week Treatment Completers With Postherpetic Neuralgia

**DOI:** 10.1155/prm/8540301

**Published:** 2026-10-12

**Authors:** Rui-Tao Shang, Qian-Qian Chen, Jia-Yi Zhang, You-Pan Lou, Ya-Nan Liu, Zi-Chuan Yue, Li-Li Zheng, Ying Huang

**Affiliations:** ^1^ Department of Pain Nanjing Drum Tower Hospital Clinical College of Nanjing University of Chinese Medicine Nanjing China nju.edu.cn; ^2^ Department of Pain Nanjing Drum Tower Hospital Affiliated Hospital of Medical School Nanjing University Nanjing China nju.edu.cn

**Keywords:** crisugabalin, efficacy, postherpetic neuralgia, pregabalin

## Abstract

**Background:**

Pregabalin (PGB) is commonly used for postherpetic neuralgia (PHN), but its clinical effectiveness may be limited by insufficient pain relief or adverse events (AEs). Crisugabalin (CGB) is a novel α2δ‐1 ligand that has demonstrated efficacy in randomized placebo‐controlled trials. However, comparative real‐world evidence for CGB and PGB remains limited.

**Methods:**

This single‐center retrospective cohort study included patients with PHN who completed at least 12 weeks of CGB or PGB monotherapy between January and June 2025. In October 2025, a single structured telephone interview was conducted to retrospectively assess patients’ recalled status at treatment initiation (T0) and at Weeks 1, 4, and 12. Outcomes included the Numerical Rating Scale (NRS), Pittsburgh Sleep Quality Index (PSQI), and the physical and mental component summary scores of the 36‐Item Short Form Health Survey (SF‐36). Linear mixed effects models (LMMs) were designated as the primary analyses and adjusted for age, sex, pain location, pre‐enrollment pain duration, hypertension, and diabetes. Repeated measures analysis of variance and the LMM analysis based on inverse probability weighting of propensity scores were performed as sensitivity analysis. Safety outcomes were descriptively assessed among treatment completers.

**Results:**

A total of 113 patients were included, including 56 in the CGB group and 57 in the PGB group. In the adjusted LMMs, significant treatment‐by‐time interactions were observed for NRS scores (*p* < 0.001), PSQI scores (*p* < 0.001), and SF‐36 mental component summary scores (*p* = 0.003), the extent to which these three scores of the CGB group improved over time may be higher than that of the PGB group. The treatment‐by‐time interactions were not statistically significant for SF‐36 physical component summary scores (*p* = 0.377). Sensitivity analyses using repeated measures analysis of variance produced concordant findings for NRS, PSQI, and SF‐36 mental component scores but not for SF‐36 physical component scores. At least one AE was reported by 11 of 56 patients in the CGB group and 18 of 57 patients in the PGB group, but there was no overall significant difference in AEs (*p* = 0.196).

**Conclusions:**

Among patients who completed 12 weeks of treatment, the CGB group was associated with statistical differences to some extent in NRS, PSQI, and SF‐36 mental component scores than PGB group, and these degree of improvement over time may be more pronounced. Due to the substantial bias in retrospective studies and the relatively small sample size, these findings should be interpreted as exploratory, merely have reference value, and require more confirmation in prospective controlled studies in the future.

## 1. Introduction

Postherpetic neuralgia (PHN) is a chronic neuropathic pain (NP) disorder that persists for ≥ 3 months after the resolution of herpes zoster rash and represents one of the most prevalent and debilitating herpes zoster’s complications. The clinical management of PHN requires a multimodal strategy to optimize pain relief. While topical agents (e.g., 5% lidocaine patches) offer localized analgesia with minimal systemic risk, and interventional procedures (e.g., nerve blocks or spinal cord stimulation) are typically reserved for refractory cases, systemic pharmacotherapy remains the preferred cornerstone for the majority of patients. Because surgical interventions inherently involve tissue damage and are rarely indicated, oral pharmacotherapy remains the modality in PHN management. Current first‐line treatments for NP primarily consist of antidepressants and anticonvulsants [1–3], including serotonin–norepinephrine reuptake inhibitors, tricyclic antidepressants, and calcium channel modulators such as gabapentin, pregabalin (PGB), and crisugabalin (CGB).

PGB is a second‐generation central nervous system calcium channel modulator that selectively binds to the voltage‐gated calcium channels’ α2‐δ subunit. Through this mechanism, PGB inhibits the excitatory neurotransmitters’ release, including norepinephrine and glutamate, thereby reducing neuronal hyperexcitability and alleviating NP. PGB has been reported to improve pain intensity and sleep‐related outcomes in Chinese PHN patients and is generally well tolerated [4, 5], with efficacy and safety profiles comparable to those observed in international populations [6]. Despite these established benefits, a substantial proportion of patients derive limited therapeutic benefit from PGB or discontinue treatment due to insufficient efficacy or tolerability concerns [7].While PGB has long been a standard treatment, its practical use is frequently limited by significant demerits, such as dose‐dependent adverse effects, most commonly dizziness and drowsiness. Additionally, prolonged administration exceeding 6 weeks has been associated with weight gain, which may negatively affect treatment adherence [8].

Comparatively, CGB is a third‐generation central nervous system calcium channel modulator that shares a similar pharmacological mechanism with PGB but differs in molecular structural design, resulting in enhanced binding affinity [9]. Notably, CGB binds to the α2‐δ subunit with approximately 23‐fold greater affinity than PGB [10]. Moreover, preclinical studies have demonstrated that CGB exerts superior therapeutic effects on hyperalgesia and allodynia compared with PGB [11]. In two rat NP models, CGB exhibited a lower minimum effective dose (MED), a longer duration of analgesic action following administration, and potentially improved central nervous system safety relative to PGB [10]. Furthermore, phase III clinical trials have shown that daily doses of 40‐ and 80‐mg CGB substantially enhanced pain outcomes in PHN and diabetic peripheral NP (DPNP) patients, with favorable tolerability and an overall acceptable safety profile [12, 13]. Nevertheless, evidence regarding the long‐term efficacy and safety of CGB in routine clinical practice remains limited, underscoring the need for additional real‐world data.

Despite these merits, research gaps remain regarding the comparative effectiveness of CGB and PGB in real‐world clinical settings. Previous clinical trials have primarily focused on pain intensity as the primary endpoint, but there is limited evidence comparing the longitudinal impact of these two agents on other critical patient outcomes, such as sleep quality and depressive symptoms. Addressing these gaps is of high clinical significance for optimizing individualized pharmacotherapy and improving the overall quality of life (QOL) for PHN patients.

Accordingly, this study aims to retrospectively analyze outpatient prescription data from real‐world clinical settings to evaluate treatment outcomes in patients with PHN. The evaluation specifically focused on changes in pain intensity, sleep quality, and physical or mental health over a 12‐week treatment period, alongside a comprehensive assessment of safety and tolerability. The findings aim to support individualized pharmacotherapy and informing evidence‐based clinical decision making.

## 2. Methods

### 2.1. Study Design and Participants

This retrospective cohort study was initiated in October 2025 at the Department of Pain Medicine, Nanjing Drum Tower Hospital. Eligible patients with PHN were identified from outpatient electronic medical records between January and June 2025. A total of 113 patients who met the selection criteria were included: 56 patients who received CGB monotherapy formed the observation group and 57 patients who received PGB monotherapy served as the control group. All patients included in this study were diagnosed with PHN for the first time and had not received other NP medications before and during treatment with PGB or CGB. Other chronic disease medications can be used normally and are not limited to antihypertensive and antidiabetic drugs.

All participants completed a 12‐week treatment period. Longitudinal outcome data, including pain intensity and QOL, were collected through structured telephone follow‐up interviews conducted in October 2025 with patients and their families to retrospectively assess outcomes at baseline: on the day of the visit prior to the start of treatment (T0), Week 1 (T1), Week 4 (T4), and Week 12 (T12). The study followed the Declaration of Helsinki and was approved by the Ethics Committee of Nanjing Drum Tower Hospital (No. 2025‐0527‐02), with informed consent waived due to its retrospective design.

### 2.2. Methods

#### 2.2.1. Data Collection

Longitudinal outcomes were retrospectively collected through structured telephone interviews conducted in October 2025 for baseline (T0), Week 1 (T1), Week 4 (T4), and Week 12 (T12). A standardized interview form was used, with treatment‐related time points serving as temporal anchors. To facilitate telephone administration, unnecessary verbatim repetition of conceptually overlapping questions was avoided, while all information required for instrument scoring was obtained. Technical terms were clarified in patient‐friendly language without altering the intended meaning or scoring criteria. Objective treatment information was verified against outpatient records, and patients unable to provide sufficiently clear recall were excluded. Some results already present in medical records still need to be collected during telephone interviews. Cases where telephone interview results differ significantly from outpatient records are excluded. All patients meeting the inclusion criteria had all the information required for this study collected during a single retrospective telephone interview. All clinical physicians participating in the telephone follow‐ups were blinded and unaware of whether the patients had received PGB or CGB.

#### 2.2.2. Limitation and Measures to Reduce Bias

All telephone follow‐ups in this study were conducted by clinical physicians from our hospital’s Pain Department with experience in telephone follow‐ups. During the telephone follow‐up period, we ensured that patients were accompanied by family members at home and in a good mental state. For questions that patients themselves could not determine, their children and partners also participated in answering to reduce bias. But family members are only permitted to assist in recalling objective dates, medication use, and observable behaviors, and must not substitute for the patient’s responses to subjective symptom items. The follow‐up duration for each patient was not less than 30 min. To facilitate recall, the start date of treatment and preset follow‐up time points were used as temporal anchors, and patients who could not provide sufficiently clear recollections of symptoms or functional status were excluded. The questionnaire was presented in a structured and patient‐friendly manner. When overlapping concepts appeared across different questionnaires, unnecessary verbatim repetition was avoided while ensuring the acquisition of all information required for scoring each scale. Technical terms or terms that may be difficult to understand were clarified using plain language, without altering the original meaning of items, response options, or scoring criteria. Furthermore, we reviewed the outpatient medical records of all patients who completed the telephone follow‐up to cross‐verify the interview results. However, due to the retrospective nature of the assessment and variations in patients’ recall intervals, substantial recall bias remains. These limitations will be further emphasized in the discussion section.

### 2.3. Selection Criteria

*Inclusion criteria were as follows*: (1) a confirmed diagnosis of PHN (defined as pain persisting for ≥ 3 months after the onset of shingles rash, ICD‐11: 6B02.2) by outpatient physicians, with a Numerical Rating Scale (NRS) score ≥ 4; (2) age ≥ 18 years; (3) receipt of either CGB or PGB, and the medication duration should be no less than 12 weeks.

*Exclusion criteria included*: (1) concomitant use of other analgesic agents for NP (e.g., gabapentin or tricyclic antidepressants); (2) severe hepatic or renal impairment, defined as a GFR < 30 mL/min; (3) pregnancy or lactation; (4) pain related to malignant disease; (5) those who took the medication for less than 12 weeks; (6) individuals who interrupt medication intake or have an interrupted treatment process due to personal or other reasons during the course of medication; (7) those who received surgical treatment during the medication period; (8) those who were hospitalized for other diseases during the medication period; (9) those who were unable to clearly recall changes in pain intensity or QOL after taking the medication.

### 2.4. Medication Protocols

Outpatient prescriptions were administered according to clinical practice. Patients received either oral PGB or oral CGB as monotherapy. Aside from this, there is no particular patient screening for the two drugs. The control group included patients who received monotherapy with oral PGB, starting at a dose of 75 mg twice daily (bid), which was gradually increased to 150 mg bid within 1 week of home medication. The observation group included patients who received monotherapy with oral CGB, since administration of CGB does not require a titration process, so patients starting at a dose of 20 mg bid; if analgesic effect was unsatisfactory when the patient returned to the outpatient clinic, the dose was increased to 40 mg bid.

Physicians in our hospital’s Pain Management Department do not prescribe medications randomly when issuing prescriptions to outpatient patients. In routine outpatient practice, the final choice between CGB and PGB is made through a discussion between the physician and patient, with appropriate consideration given to physician recommendations, patient preferences, prior knowledge or treatment experience, and applicable institutional prescription policies. However, due to insufficient clinical evidence, there exists mild variability in selection within the range of patients with comparable physical conditions: (1) Patients with a history of PGB use who have demonstrated good analgesic tolerance continue to receive PGB. (2) First‐time users of gabapentinoids are influenced by the physician’s personal preference, with different physicians tending to prioritize PGB or CGB. (3) Some physicians indeed exhibit a tendency to prescribe the newer medication CGB more frequently to patients with longer disease duration. (4) Patients with inadequate analgesic effect or poor tolerance to PGB are switched by physicians to CGB, but all patients who replaced medications during the treatment period were not included in the study.

### 2.5. Outcome Measures

1. Analgesic Efficacy: The analgesic efficacy of the two treatment regimens in PHN patients was evaluated using the NRS [14]. The NRS is a validated instrument that quantifies subjective pain intensity on a 0–10 scale, where 0 indicates no pain, and 1–3, 4–6, and 7–10 represent mild, moderate, and severe pain, respectively. Pain intensity was assessed at four predefined time points: baseline (Day 1, T0), Week 1 (T1), Week 4 (T4), and Week 12 (T12).
2. Sleep Quality: Assessed utilizing the Pittsburgh Sleep Quality Index (PSQI) [15]. The PSQI comprises seven components, including 19 self‐rated items and 5 observer‐rated items, yielding a total score ranging from 0 to 21, with higher scores indicating poorer sleep quality. Clinical grading was defined as follows: 0–5, very good sleep quality; 6–10, relatively good sleep quality; 11–15, fair sleep quality; and 16–21, poor sleep quality.
3. QOL: Evaluated using the 36‐Item Short Form Health Survey (SF‐36) [16]. The SF‐36 includes eight independent domains, each scored on a scale from 0 to 100, with lower scores showing poorer health. In this study, analyses focused on two core domains: physical health and mental health.
4. Safety: Safety outcomes were evaluated by statistically analyzing the occurrence of adverse reactions during treatment, including dizziness, drowsiness, nausea, constipation, vomiting, fatigue, peripheral edema, and itching. The incidence rates of these adverse events (AEs) were calculated and compared between the two treatment groups.
5. Confounding Factor Control: Due to the interference of numerous confounding factors in retrospective studies, this study incorporated a total of six confounding factors as covariates, including gender, age, pain location, pre‐enrollment pain duration, and hypertension and diabetes [17].

### 2.6. Statistical Analysis and Control of Confounding Factors

Data normality was evaluated utilizing the D’Agostino–Pearson test. Continuous variables following a normal distribution are presented as the mean (standard deviation [$\overset{¯}{\text{x}}$], SD). Continuous variables following a non‐normal distribution are expressed as the interquartile range (IQR). Categorical variables are reported as frequencies and percentages and were compared using the chi‐square test or Fisher’s exact test, depending on expected cell counts.

This study employed three parallel methods, linear mixed effects models (LMMs), repeated measures analysis of variance (RM‐ANOVA), and LMM based on inverse probability weighting using propensity scores (IPTW‐LMM) to validate four longitudinal outcome measures (NRS, PSQI, SF‐36 physical and mental component summaries). LMM and RM‐ANOVA were analyzed using SPSS version 30, while IPTW‐LMM was analyzed using The R Project for Statistical Computing version 4.4.1.

(1) LMM: Medication, time, and their interaction term were treated as fixed effects. Age, gender, pain location, pre‐enrollment pain duration, hypertension, and diabetes were included as covariates. Patients were modeled as random intercepts, with a diagonal structure for the repeated measures covariance matrix. Results of multiple comparisons were adjusted using hierarchical Bonferroni correction to reduce Type I error. (2) Repeated Measures Analysis of Variance: Designed as intercepts, medication, age, gender, pain location, pre‐enrollment pain duration, hypertension, diabetes. The analytical result is also medication, time, and their interaction effects. Post hoc tests for between‐subject effects were adjusted for all baseline confounding variables using Bonferroni correction. (3) LMM Based on Inverse Probability Weighting Using Propensity Scores: The same covariates as before are used to calculate propensity scores. Stabilized weights are adopted, and the weights are truncated at the 1%–99% range to avoid extreme weights. The stabilized weights are incorporated into the LMM to obtain the IPTW‐LMM weighted model. Standardized mean differences (SMD) are used to evaluate covariate balance before and after weighting. A good baseline balance is considered achieved when |SMD| < 0.1.

A hierarchical Bonferroni correction strategy for multiple testing correction is adopted, with each scale as an independent hierarchy. Correction is applied only to multiple comparisons within the same scale, while no correction is performed between different scales. For intergroup comparisons within a single scale, *k* = 4, and the corrected *p* value is the initial *p* value multiplied by 4. LMM was used as the primary analysis, IPTW‐LMM and RM‐ANOVA were used solely for sensitivity analysis. When the tendencies of three analytical results are inconsistent, the LMM shall be taken as the primary basis for explanation. All statistical tests were two sided, and a corrected *p* < 0.05 was considered statistically significant.

## 3. Results

### 3.1. Patients Characteristics

A total of 113 patients completed telephone follow‐up after the start of the study and were included in this analysis. And, among them, 56 patients who received CGB monotherapy comprised the observation group, while 57 patients who received PGB monotherapy constituted the control group. All participants completed a standardized 12‐week telephone follow‐up prior to data analysis. Outcome assessments were conducted at baseline (T0), Week 1 (T1), Week 4 (T4), and Week 12 (T12). As shown in Table 1, baseline characteristics, including sex (male: 51.8% vs. 56.1%, *p* = 0.707), age (67.63 ± 13.08 vs. 69.14 ± 11.80 years, *p* = 0.519), pain location (*p* = 0.390), hypertension (*p* > 0.999), diabetes mellitus (*p* = 0.315), baseline NRS scores (5.86 ± 1.17 vs. 6.09 ± 1.18, *p* = 0.299), baseline PSQI scores (14.36 ± 2.75 vs. 14.58 ± 3.07, *p* = 0.687), baseline HAMD‐17 scores (16.88 ± 6.97 vs. 17.72 ± 7.05, *p* = 0.523), baseline SF‐36 Physica scores (41.38 ± 4.45 vs. 40.28 ± 5.92, *p* = 0.269), and baseline SF‐36 mental scores (39.00 ± 6.68 vs. 37.28 ± 6.03, *p* = 0.154) were comparable between the two groups, with no statistically significant differences observed. No intolerable or severe adverse reactions occurred in either group during the period of taking medication at home in the past. Notably, the *p* value for pre‐enrollment pain duration at baseline was very close to the 0.05 critical value (*p* = 0.055) (Table 1). A clinically relevant imbalance was observed in pre‐enrollment pain duration, because all five patients with pain lasting at least 12 months were in the CGB group. This case was caused by a bias where outpatient physicians were more inclined to prescribe CGB to patients with pain duration exceeding 1 year, it had played an indispensable role in the subsequent confounding factor analysis.

**TABLE 1:** General patient information.

Characteristics	CGB group (*n* = 56)	PGB group (*n* = 57)	*p*
Sex, *n* (%)			
Male	29 (51.8%)	32 (56.1%)	0.707
Female	27 (48.2%)	25 (43.9%)	
Age, mean (SD), years	67.63 (13.08)	69.14 (11.80)	0.519
PHN location, *n* (%)			
Limbs	9 (16.1%)	9 (15.8%)	0.390
Chest and back	28 (50.0%)	24 (42.1%)	
Abdomen	13 (23.2%)	11 (19.3%)	
Others	6 (10.7%)	13 (22.8%)	
Duration of pain before enrollment (month)			
3 < *t* ≤ 6	48 (85.7%)	55 (96.5%)	0.055
6 < *t* ≤ 12	3 (5.4%)	2 (3.5%)	
12 ≤ *t*	5 (8.9%)	0 (0)	
Hypertension			
Have	17 (30.4%)	17 (29.8%)	> 0.999
No	39 (69.6%)	40 (70.2%)	
Diabetes mellitus			
Have	11 (19.6%)	7 (12.3%)	0.315
No	45 (80.4%)	50 (87.7%)	
Scale			
NRS scores T0, mean (SD)	5.86 (1.17)	6.09 (1.18)	0.299
PSQI scores T0, mean (SD)	14.36 (2.75)	14.58 (3.07)	0.687
SF‐36 Physical scores T0, mean (SD)	41.38 (4.45)	40.28 (5.92)	0.269
SF‐36 Mental scores T0, mean (SD)	39.00 (6.68)	37.28 (6.03)	0.154

*Note:* Data are mean (SD) or *n* (%).

### 3.2. Results of LMMs

#### 3.2.1. NRS

The main effect of treatment was significant (*F* [1, 441.05] = 104.03, *p* < 0.001), with Group CGB showing a significantly lower overall NRS score than Group PGB (estimate = −1.820, 95% CI [−2.382, −1.258], *p* < 0.001). The main effect of time was also significant (*F* [3, 198.72] = 166.18, *p* < 0.001), indicating that NRS scores decreased significantly over time in both groups. A significant interaction effect between medication and time was observed (*F* [3, 198.72] = 7.34, *p* < 0.001), suggesting that the downward trends differed significantly between the two groups. Pain location (*F* [3, 437.25] = 7.30, *p* < 0.001), hypertension (*F* [1, 437.25] = 9.01, *p* = 0.003), and diabetes (*F* [1, 437.25] = 8.19, *p* = 0.004) had significant effects on NRS scores; however, gender, pre‐enrollment pain duration, and age did not show significant effects (all *p*s > 0.05) (Table 2).

**TABLE 2:** LMM results.

Effect	Molecular df	Denominator df	*F*	*p*
NRS				
Drug	1	441.053	104.035	< 0.001
Time	3	198.719	166.181	< 0.001
Drug × Time	3	198.719	7.342	< 0.001
Age	1	437.246	0.038	0.846
Gender	1	437.246	0.498	0.481
Pain location	3	437.246	7.296	< 0.001
Duration of pain before enrollment	2	437.246	2.572	0.078
Hypertension	1	437.246	9.007	0.003
Diabetes mellitus	1	437.246	8.195	0.004
PSQI				
Drug	1	446.078	75.528	< 0.001
Time	3	201.156	95.460	< 0.001
Drug × Time	3	201.156	6.574	< 0.001
Age	1	444.410	0.655	0.419
Gender	1	444.410	10.279	0.001
Pain location	3	444.410	2.786	0.040
Duration of pain before enrollment	2	444.410	2.607	0.075
Hypertension	1	444.410	4.094	0.044
Diabetes mellitus	1	444.410	4.675	0.031
SF‐36 physical				
Drug	1	444.910	31.793	< 0.001
Time	3	203.556	30.371	< 0.001
Drug × Time	3	203.556	1.037	0.377
Gender	1	443.011	34.338	< 0.001
Pain location	3	443.011	0.980	0.402
Duration of pain before enrollment	2	443.011	6.323	0.002
Hypertension	1	443.011	0.048	0.827
Diabetes mellitus	1	443.011	0.370	0.543
SF‐36 mental				
Drug	1	443.012	94.893	< 0.001
Time	3	205.008	49.894	< 0.001
Drug × Time	3	205.008	4.797	0.003
Age	1	438.992	7.620	0.006
Gender	1	438.992	10.118	0.002
Pain location	3	438.992	0.693	0.557
Duration of pain before enrollment	2	438.992	2.988	0.051
Hypertension	1	438.992	4.292	0.039
Diabetes mellitus	1	438.992	2.506	0.114

Pairwise comparisons of time points (with Bonferroni correction) revealed that all pairwise differences were significant (all *p*s < 0.001). In Group CGB, NRS scores decreased from 6.208 ± 0.207 at T0 to 1.851 ± 0.242 at T12 (a reduction of 4.357 points). In Group PGB, scores decreased from 6.618 ± 0.223 at T0 to 3.670 ± 0.255 at T12 (a reduction of 2.948 points). The magnitude of decrease in Group CGB was significantly greater than that in Group PGB. Covariance parameters indicated that residual variances at each time point were significant (all *p*s < 0.001) (Supporting Tables S1 and S2).

#### 3.2.2. PSQI

The main effect of medication was significant (*F* [1, 446.08] = 75.53, *p* < 0.001), with Group CGB showing significantly lower PSQI scores than Group PGB (estimate = −3.816, 95% CI [−5.130, −2.503], *p* < 0.001). The main effect of time was also significant (*F* [3, 201.16] = 95.46, *p* < 0.001), with PSQI scores decreasing significantly over time in both groups. A significant interaction effect between medication and time was observed (*F* [3, 201.16] = 6.57, *p* < 0.001), indicating a significant difference in the decline trends between the two groups. Gender (*F* [1, 444.41] = 10.28, *p* = 0.001), pain location (*F* [3, 444.41] = 2.79, *p* = 0.040), hypertension (*F* [1, 444.41] = 4.09, *p* = 0.044), and diabetes (*F* [1, 444.41] = 4.68, *p* = 0.031) had significant effects on PSQI scores; pre‐enrollment pain duration and age did not have significant effects (all *p*s > 0.05) (Table 2).

Bonferroni‐corrected pairwise comparisons revealed significant differences between all time points (all *p*s < 0.01). Group CGB scores decreased from 15.046 ± 0.511 at T0 to 6.796 ± 0.570 at T12 (a reduction of 8.250 points), while Group PGB scores decreased from 15.647 ± 0.549 at T0 to 10.612 ± 0.603 at T12 (a reduction of 5.035 points), with Group CGB showing a significantly greater reduction. Covariance parameters indicated that residual variances at each time point were significant (all *p*s < 0.001) (Supporting Tables S3 and S4).

#### 3.2.3. SF‐36 Physical Assessment

The main effect of treatment was significant (*F* [1, 444.91] = 31.79, *p* < 0.001), with Group CGB showing significantly higher SF‐36 physical component scores than Group PGB (estimate = 3.565, 95% CI [1.492, 5.638], *p* = 0.001). The main effect of time was also significant (*F* [3, 203.56] = 30.37, *p* < 0.001), with SF‐36 physical component scores increasing significantly over time in both groups. The interaction effect between medication and time was not significant (*F* [3, 203.56] = 1.04, *p* = 0.377), indicating no significant difference in the upward trends between the two groups. Gender (*F* [1, 443.01] = 34.34, *p* < 0.001), pre‐enrollment pain duration (*F* [2, 443.01] = 6.32, *p* = 0.002), and age (*F* [1, 443.01] = 14.81, *p* < 0.001) had significant effects on SF‐36 physical component scores; the effects of pain location, hypertension, and diabetes were not significant (all *p*s > 0.05) (Table 2).

Bonferroni‐corrected pairwise comparisons revealed significant differences between T0 and T1 (difference = −2.493, *p* = 0.001), T0 and T4 (difference = −4.451, *p* < 0.001), T0 and T12 (difference = −6.231, *p* < 0.001), T1 and T4 (difference = −1.958, *p* = 0.010), and T1 and T12 (difference = −3.738, *p* < 0.001). No significant difference was found between T4 and T12 (difference = −1.780, *p* = 0.059). Group CGB scores increased from 39.883 ± 0.841 at T0 to 47.205 ± 0.896 at T12 (an increase of 7.322 points), while Group PGB scores increased from 38.500 ± 0.896 at T0 to 43.640 ± 0.947 at T12 (an increase of 5.140 points). Although Group CGB exhibited a greater magnitude of increase than Group PGB, the interaction effect did not reach statistical significance. Covariance parameters indicated that residual variances at each time point were significant (all *p*s < 0.001) (Supporting Tables S5 and S6).

#### 3.2.4. SF‐36 Mental Assessment

A significant main effect of treatment was observed (*F* [1, 443.01] = 94.89, *p* < 0.001), with Group CGB exhibiting significantly higher SF‐36 mental scores than Group PGB (estimate = 8.403, 95% CI [5.722, 11.084], *p* < 0.001). A significant main effect of time was also found (*F* [3, 205.01] = 49.89, *p* < 0.001), indicating that SF‐36 mental scores increased significantly over time in both groups. The interaction effect between medication and time was significant (*F* [3, 205.01] = 4.80, *p* = 0.003), suggesting a significant difference in the upward trends between the two groups. Gender (*F* [1, 438.99] = 10.12, *p* = 0.002), hypertension (*F* [1, 438.99] = 4.29, *p* = 0.039), and age (*F* [1, 438.99] = 7.62, *p* = 0.006) had significant effects on SF‐36 mental scores; however, pain location, pre‐enrollment pain duration, and diabetes did not (all *p*s > 0.05) (Table 2).

Bonferroni‐corrected pairwise comparisons revealed significant differences between all time points (all *p*s < 0.05). Group CGB scores increased from 37.346 ± 1.071 at T0 to 50.542 ± 1.155 at T12 (an increase of 13.196 points), while Group PGB scores increased from 35.069 ± 1.142 at T0 to 42.139 ± 1.219 at T12 (an increase of 7.070 points), with Group CGB demonstrating a significantly greater increase than Group PGB. Covariance parameters indicated that residual variances at each time point were significant (all *p*s < 0.001) (Supporting Tables S7 and S8).

### 3.3. Results of Repeated Measures Analysis of Variance

Mauchly’s sphericity test indicated violation of the sphericity assumption for all (all *p*s < 0.001) (Supporting Table S9), since the Greenhouse–Geisser (GG) correction was applied to the degrees of freedom for within‐subject effects. Post hoc tests were conducted after adjustment with Bonferroni correction, the results of the RM‐ANOVA showed that, except for the main effect of time on NRS (*p* = 0.054), statistically significant differences were present for the main drug effect, time effect, and the interaction effect between medication and time of all variables (Table 3). It is noteworthy that the interaction effects between medication and time for the scale indicators NRS, PSQI, and SF‐36 mental were consistent with the results of the LMM, whereas the interaction effects between medication and time for SF‐36 physical was inconsistent with the LMM results. The LMM results were selected as the reference at last.

**TABLE 3:** RM‐ANOVA results.

Effect	df	MS	*F*	*p*	Partial *η* ^2^
NRS					
Drug	(1102)	188.477	38.625	< 0.001	0.275
Time	(1.985, 202.439)	3.737	2.971	0.054	0.028
Time × Drug	(1.985, 202.439)	24.074	19.139	< 0.001	0.158
PSQI					
Drug	(1102)	773.055	25.421	< 0.001	0.200
Time	(1.742, 177.721)	23.597	3.316	0.045	0.031
Time × Drug	(1.742,177.721)	137.298	19.295	< 0.001	0.159
SF‐36 physical					
Drug	(1102)	777.812	8.649	0.004	0.078
Time	(1.842, 187.845)	32.134	3.430	0.038	0.033
Time × Drug	(1.842, 187.845)	45.236	4.829	0.011	0.045
SF‐36 mental					
Drug	(1102)	3844.067	32.899	< 0.001	0.244
Time	(1.461, 149.022)	146.665	3.933	0.034	0.037
Time × Drug	(1.461, 149.022)	445.624	11.949	< 0.001	0.105

### 3.4. Results of LMM Based on Inverse Probability Weighting Using Propensity Scores

The results of the LMM analysis based on inverse probability weighting of propensity scores showed that the weighted models for all measured indicators were consistent in direction and significant (*p* < 0.05) with the unweighted models in terms of interaction term coefficients, indicating that the LMM results are less affected by confounding factors and possess sufficient stability (Table 4).

**TABLE 4:** IPTW‐LMM results.

Effect	Interaction coefficient	SMD	*p*
NRS			
Weighted model	1.159	0.254	< 0.001
Unweighted model	0.985	0.251	< 0.001
PSQI			
Weighted model	2.543	0.549	< 0.001
Unweighted model	2.172	0.553	< 0.001
SF‐36 physical			
Weighted model	−1.709	0.643	0.008
Unweighted Model	−1.372	0.640	0.033
SF‐36 mental			
Weighted model	−4.932	1.234	< 0.001
Unweighted model	−4.107	1.167	< 0.001

### 3.5. AEs

Safety assessments, summarized in Table 5, showed that both treatments were well tolerated, with no serious AEs recorded. Although a lower total number of AEs occurred in the CGB group (*n* = 14) compared to the PGB group (*n* = 26), and the total number of patients at least occurred once experiencing AEs in the CGB group (*n* = 11) was also fewer than that in the PGB group (*n* = 18), the incidence of specific events was generally comparable (*p* = 0.196). Dizziness was the most common AE in both cohorts, affecting 12% of patients in each group (*p* > 0.999). Notably, the incidence of drowsiness was lower in the CGB group (2%) compared to the PGB group (12%), but it still does not have statistical significance (*p* = 0.061). Other AEs, such as nausea, constipation, and edema, occurred at low and comparable rates between the two groups (all *p*s > 0.05).

**TABLE 5:** Safety data.

Adverse events	CGB group (*n* = 56)	PGB group (*n* = 57)	*p*
Total number of individuals who experienced adverse events	11 (19.6%)	18 (31.6%)	0.196
Total number of individuals who did not experience adverse events	45 (80.4%)	39 (68.4%)	

Dizziness	7 (12%)	7 (12%)	> 0.999

Drowsiness	1 (2%)	7 (12%)	0.061

Nausea	2 (4%)	3 (5%)	> 0.999

Constipation	2 (4%)	4 (7%)	0.679

Vomiting	1 (2%)	0 (0%)	0.496

Fatigue	0 (0%)	2 (4%)	0.496

Edema	1 (2%)	1 (2%)	> 0.999

Itching	0 (0%)	2 (4%)	0.496

*Note:* Data are *n* (%).

## 4. Discussion

PHN, the most common neuropathic complication of herpes zoster, not only causes persistent pain but also induces uncomfortable symptoms such as paresthesia, sleep disturbances, and emotional distress. The course of PHN can last for several months, and in some cases, symptoms may persist long term or even lifelong, posing significant challenges for clinical management. Although multiple pharmacological options are available, many patients develop reduced responsiveness to commonly used traditional therapies, leading to suboptimal pain control, and in some cases, this diminished therapeutic response may persist for years [18].

Current treatment options for PHN include calcium channel modulators, topical therapies such as 5% lidocaine and 8% capsaicin patches, and opioids like hydromorphone, all of which have shown some efficacy in pain relief and QOL improvement [19–21]. However, opioids carry substantial risks including addiction, withdrawal, and serious adverse effects, and are generally reserved for patients with severe, refractory pain. Topical treatments may cause local reactions such as erythema, swelling, or burning, and require specific application techniques, limiting their convenience and long‐term use. Novel targeted therapies, such as adaptor‐associated kinase 1 (AAK1) inhibitors (e.g., LX‐9211), are under investigation, but further clinical evidence is needed [22]. In this context, calcium channel modulators continue to represent a cornerstone of first‐line pharmacotherapy for PHN. In relevant studies on the dose equivalence transition from gabapentin to PGB, gabapentin and PGB have an approximate conversion ratio of 6:1 [23]. In a meta‐analysis regarding PGB and gabapentin that had included 14 randomized controlled trials and 3545 participants, there is substantial evidence supporting PGB’s superior efficacy over gabapentin for PHN, albeit with a trade‐off of reduced safety profile. However, further support from clinical randomized trials remains necessary [24]. Currently, clinical research on the dose equivalence relationship between CGB and PGB remains highly insufficient. Although CGB demonstrated good tolerability at both 40 and 80 mg/d doses in Phase III clinical randomized trials for the treatment of PHN [12], and showed earlier analgesic onset than 300 mg/d PGB in a controlled study for DPNP [25], more controlled studies are still needed.

Although both CGB and PGB are calcium channel modulators, they may differ in analgesic efficacy and safety profile. In the analysis results of this study, the main drug effect and the drug–time interaction were significant between the CGB group and the PGB group. At the same time point, the NRS scores of the CGB group were significantly lower than those of the PGB group, and the degree of decline over time was more pronounced. However, the presence of recall bias reduces the quality of the data, these exploratory results merely can be indicating that CGB may have further research value for its primary efficacy in relieving PHN. Significant differences in baseline and treatment period pain levels were observed among patients with different pain locations. Patients with comorbid hypertension and diabetes exhibited significantly higher pain levels. Given that these three confounding factors had a significant impact on NRS scores, this diminished the strength of the results. Beyond PHN, CGB has also demonstrated therapeutic potential in diabetic peripheral neuropathic pain (DPNP), achieving effective pain relief more rapidly than PGB during a 14‐day pretreatment period, further supporting its broad analgesic efficacy [25].

In terms of safety, dizziness was the most commonly reported AE in both groups, consistent with the known pharmacological profile of α2δ ligands. Although the total number of AEs was slightly lower in the CGB group, incidences of dizziness, drowsiness, nausea, constipation, vomiting, fatigue, peripheral edema, and itching were generally similar between groups. Although the total number of AEs in the CGB group less than the PGB group, there was no significant difference in the number of patients who experienced AEs. Due to the small sample size and multiple confounding factors in this study, this little difference does not provide clinical security conclusion and may holds reference for further large‐scale research. Notably, drowsiness occurred less frequently in the CGB group. Dizziness and drowsiness belong to the same category of central nervous system adverse effects; however, these two adverse reaction symptoms do not necessarily occur simultaneously, and there is individual variation among the population. In both groups, drowsiness showed *p* > 0.05, indicating no statistical difference. Furthermore, due to the presence of patient recall bias and limitations of sample size, it cannot be concluded that there is any distinction between the two groups. Weight gain, a well‐documented adverse effect of PGB, was not assessed in this study due to the retrospective design and the lack of reliably recorded body weight data. We considered whether to collect patients’ weight data. However, we could not determine if patients’ diet, exercise, and other lifestyle habits had undergone significant changes during the medication period following PHN diagnosis, as diet and other habits are also decisive factors influencing weight. Due to the numerous life factors that influence patients’ weight changes, and in the absence of critical lifestyle statistics, the accuracy of weight data would be the lowest. Therefore, we did not collect these data, which represents an insufficiency in the safety aspect of this study. In addition, due to the small sample size of this study, it is insufficient to observe rare or more severe AEs, and thus cannot demonstrate any clinical level of safety. Overall, no significant differences in safety were found between CGB and PGB in this study, with both drugs generally well tolerated and no serious AEs reported. These results are consistent with findings from Phase III clinical trials evaluating CGB for PHN [12]. Moreover, preclinical drug withdrawal studies have demonstrated minimal physical dependence on CGB in rodent models, suggesting a lower potential for drug abuse and indicating that CGB may represent a safer alternative to PGB and mirogabalin [26].

In addition to severe and persistent pain, PHN profoundly affects multiple aspects of patients’ daily lives, including emotional well‐being, physical functioning, sleep, routine activities, and engagement in hobbies [27]. Among these domains, sleep quality is often the most severely disrupted [28] and contributes substantially to the overall disease burden and associated economic costs. Existing evidence indicates that PGB and gabapentin can improve sleep quality in patients with NP primarily through pain reduction [29], with PGB demonstrating superior effects on sleep quality in PHN compared with gabapentin [28]. However, the impact of CGB on sleep quality and QOL has been insufficiently explored in prior studies. In the results of this study, both the main drug effect and the drug–time interaction effect between the CGB group and the PGB group were statistically significant. Within the same time points, the CGB group exhibited a greater downward trend in PSQI scores compared to the PGB group, and the degree of decline over time was more pronounced. However, due to the inherent limitations of recall bias and small sample size, this can only indicate that CGB may have some value worth in‐depth research for improving sleep quality, and cannot support any clinical conclusions. During the previous treatment period, pain location, hypertension, and diabetes still exerted a significant influence on PSQI scores. Notably, the effect of gender on PSQI scores was also non‐negligible, with males having an estimated average PSQI score that was 0.995 points lower than females (Supporting Table S3). This difference does not indicate that gabapentinoids provide a lesser improvement in sleep quality for female PHN patients; rather, it suggests the possibility for further research. At the mechanistic level, previous molecular and neurobiological studies have shown that PGB can restore sleep architecture in mouse models of spared nerve injury by enhancing power in the 3.5–5.5 Hz frequency band during rapid eye movement sleep and increasing sleep spindle formation during transitions between sleep stages, thereby normalizing the expression of circadian rhythm‐related genes [30]. In contrast, the biological mechanisms underlying the sleep‐promoting effects of CGB remain largely unexplored and warrant further investigation in future experimental and clinical studies.

Multiple factors contribute to impaired QOL in patients with PHN, including pain severity, medication related adverse effects, and sleep disturbance. In the analysis results, for the SF‐36 physical health assessment, the main effect of medication was significant, with the SF‐36 physical component summary scores of the CGB group being higher than those of the PGB group at the same time points. However, the medication–time interaction effect was not significant, indicating there was no significant difference in the change over time of the SF‐36 physical scores between the two groups. It should be noted that gender, pre‐enrollment pain duration, and age had a significant impact on the SF‐36 physical component score. Male participants scored an average of 2.836 points higher than female participants (Supporting Table S7). For the SF‐36 mental health assessment, both the main effect of medication and the interaction effect of medication–time were significant. The CGB group had higher scores at the same time points, with a more pronounced upward trend compared to the PGB group. Gender, hypertension, and age had significant effects on SF‐36 mental component scores. Male participants still scored an average of 1.971 points higher than female participants (Supporting Table S9). Generally, patients with greater age had lower scores. Given the numerous confounding factors and inherent limitations such as recall bias in this study, these results can only indicate that CGB may hold potential significance for further research on improving the QOL of PHN patients. They do not address any clinical issues and merely provide reference value.

Emerging evidence also indicates that α2δ ligands may influence immune‐related processes. For example, immunological studies have shown that PGB treatment increases the ratio of helper T cells and elevates interleukin‐17 levels in patients with PHN, suggesting a potential immunomodulatory effect [31]. These observations provide a novel perspective for future investigations into the pharmacological mechanisms of CGB. In patients with refractory PHN, combination therapy with PGB and hydromorphone has been reported to yield improved pain control and sleep quality, with relatively mild adverse effects, primarily dizziness and nausea, and no evidence of respiratory depression [32]. Given the limitations of monotherapy, further clinical studies are warranted to evaluate the therapeutic value of CGB as part of combination treatment strategies.

As a small‐sample retrospective study employing telephone follow‐up, this research is subject to substantial recall bias and random factors, resulting in low data reliability. Due to the tendency of physicians at our hospital to prioritize prescribing CGB for patients with pain lasting more than 1 year in outpatient settings, this has affected the stability of the baseline and further reduced the reliability of the results. Despite the inclusion of six confounding factors as covariates, numerous unmeasured and residual confounding effects that were difficult to control diminished the strength of the results. All patient samples included in this study were those who completed 12 weeks of monotherapy, a selection bias that cannot account for individuals with shorter (< 12 weeks) or longer treatment durations. The small sample size was insufficient to detect rare AEs, leading to greater influence from patient‐specific variability and lower reference strength for safety data. In the PGB group of this study, only a single dose of 300 mg/day was utilized, and a dose equivalence design was not established. Furthermore, the interpretation of all results from this study is influenced by our single‐center setting and cannot represent populations from other regions, ethnicities, or countries with different climates, lifestyles, and disease prevalence rates. Accordingly, the observed associations should be regarded as hypothesis generating and require confirmation in prospective, adequately powered controlled studies.

## 5. Conclusion

Among patients who completed 12 weeks of treatment, the CGB group was associated with statistical differences to some extent in NRS, PSQI, and SF‐36 mental component scores than PGB group, and these degree of improvement over time may be more pronounced. Due to the substantial bias in retrospective studies and the relatively small sample size, these findings should be interpreted as exploratory, merely have reference value, and require more confirmation in prospective controlled studies in the future.

## Author Contributions

Ying Huang, Li‐Li Zheng, and Zi‐Chuan Yue were involved in the development and design of the study concept. Rui‐Tao Shang contributed to the initial manuscript writing. Ya‐Nan Liu and You‐Pan Lou were involved in intervention and assessment. Qian‐Qian Chen, Jia‐Yi Zhang, and Rui‐Tao Shang were involved in data acquisition and analysis. All authors revised.

## Funding

This work was supported by the Medical Science and Technology Development Project of Nanjing (no. ZKX23022) and Jiangsu Commission of Health Scientific Research Project (no. M2024089). The authors are indebted to and thank all patients for participating in this study.

## Disclosure

All authors agreed to the final version of this article.

## Conflicts of Interest

The authors declare no conflicts of interest.

## Supporting Information

Additional supporting information can be found online in the Supporting Information section.

## Supporting information


**Supporting Information** Supporting Tables S1–S9 are provided in the Supporting Information.

## Data Availability

The data that support the findings of this study are available from the corresponding author upon reasonable request.

## References

[1] Fornasari D. , Pharmacotherapy for Neuropathic Pain: A Review, Pain and Therapy. (2017) 6, no. Suppl 1, 25–33, 10.1007/s40122-017-0091-4. PMC5701897 29178034

[2] Nalamachu S. and Morley-Forster P. , Diagnosing and Managing Postherpetic Neuralgia, Drugs & Aging. (2012) 29, no. 11, 863–869, 10.1007/s40266-012-0014-3. PMC3693437 23038608

[3] Argoff C. E. , Review of Current Guidelines on the Care of Postherpetic Neuralgia, Postgraduate Medical Journal. (2011) 123, no. 5, 134–142, 10.3810/pgm.2011.09.2469. 21904096

[4] Liu Q. , Chen H. , Xi L. et al., A Randomized, Double-Blind, Placebo-Controlled Trial to Evaluate the Efficacy and Safety of Pregabalin for Postherpetic Neuralgia in a Population of Chinese Patients, Pain Practice. (2017) 17, no. 1, 62–69, 10.1111/papr.12413. 26714731

[5] Guan Y. , Ding X. , Cheng Y. et al., Efficacy of Pregabalin for Peripheral Neuropathic Pain: Results of an 8-Week, Flexible-Dose, Double-Blind, Placebo-Controlled Study Conducted in China, Clinical Therapeutics. (2011) 33, no. 2, 159–166, 10.1016/j.clinthera.2011.02.007. 21444113

[6] Parsons B. , Pan X. , Xie L. , Chen Y. , Ortiz M. , and Whalen E. , Comparison of the Efficacy and Safety of Pregabalin for Postherpetic Neuralgia in Chinese and International Patients, Journal of Pain Research. (2018) 11, 1699–1708, 10.2147/jpr.s157856. PMC6126478 30214280

[7] Derry S. , Bell R. F. , Straube S. , Wiffen P. J. , Aldington D. , and Moore R. A. , Pregabalin for Neuropathic Pain in Adults, Cochrane Database of Systematic Reviews. (2019) 1, no. 1. 10.1002/14651858.CD007076.pub3 PMC6353204 30673120

[8] Arnold L. M. , McCarberg B. H. , Clair A. G. et al., Dose-Response of Pregabalin for Diabetic Peripheral Neuropathy, Postherpetic Neuralgia, and Fibromyalgia, Postgraduate Medical Journal. (2017) 129, no. 8, 921–933, 10.1080/00325481.2017.1384691. 28967801

[9] Alcántara Montero A. , Is Crisugabalin Signaling the Arrival of a Third Generation of α2δ Ligands?, CNS Drugs. (2026) . 10.1007/s40263-026-01292-x 41936716

[10] Gou X. , Yu X. , Bai D. et al., Pharmacology and Mechanism of Action of HSK16149, A Selective Ligand of α2δ Subunit of Voltage-Gated Calcium Channel With Analgesic Activity in Animal Models of Chronic Pain, Journal of Pharmacology and Experimental Therapeutics. (2021) 376, no. 3, 330–337, 10.1124/jpet.120.000315. 33293377

[11] Shi Z. , Chen L. , Shi S. et al., New Tricyclic γ-Aminobutyric Acid Analogue HSK16149: A Ca(2+) Channel α(2)-δ Ligand for Treating Neuropathic Pain, ACS Medicinal Chemistry Letters. (2025) 16, no. 11, 2215–2223, 10.1021/acsmedchemlett.5c00336. PMC12621040 41257002

[12] Zhang D. , Lei T. , Qin L. et al., Efficacy and Safety of Crisugabalin (HSK16149) in Adults With Postherpetic Neuralgia: A Phase 3 Randomized Clinical Trial, JAMA Dermatology. (2024) 160, no. 11, 1182–1191, 10.1001/jamadermatol.2024.3410. PMC11581609 39320907

[13] Guo X. , Zhang T. , Yuan G. et al., GABA Analogue HSK16149 in Chinese Patients With Diabetic Peripheral Neuropathic Pain: A Phase 3 Randomized Clinical Trial, JAMA Network Open. (2024) 7, no. 8, 10.1001/jamanetworkopen.2024.25614. PMC11333976 39158916

[14] Krebs E. E. , Carey T. S. , and Weinberger M. , Accuracy of the Pain Numeric Rating Scale as a Screening Test in Primary Care, Journal of General Internal Medicine. (2007) 22, no. 10, 1453–1458, 10.1007/s11606-007-0321-2. PMC2305860 17668269

[15] Buysse D. J. , Reynolds C. F.3rd, Monk T. H. , Berman S. R. , and Kupfer D. J. , The Pittsburgh Sleep Quality Index: A New Instrument for Psychiatric Practice and Research, Psychiatry Research. (1989) 28, no. 2, 193–213, 10.1016/0165-1781(89)90047-4. 2748771

[16] Ware J. E.Jr. and Sherbourne C. D. , The MOS 36-Item Short-Form Health Survey (SF-36). I. Conceptual Framework and Item Selection, Medical Care. (1992) 30, no. 6, 473–483. 1593914

[17] Wang J. , Tao R. , Jiang Y. , Ma Z. , and Xia L. , Risk Factors for Postherpetic Neuralgia: A Meta-Analysis Based on Demographic, Clinical Features, and Treatment Characteristics, Frontiers in Immunology. (2025) 16, 10.3389/fimmu.2025.1667364. PMC12521459 41103428

[18] Jeon Y. H. , Herpes zoster and Postherpetic Neuralgia: Practical Consideration for Prevention and Treatment, Korean Journal of Pain. (2015) 28, no. 3, 177–184, 10.3344/kjp.2015.28.3.177. PMC4500781 26175877

[19] Guo Z. , Xia Y. , and Zhang Z. , Efficacy and Safety of Different Medications Compared for the Treatment of Postherpetic Neuralgia: A Network Meta-Analysis, Frontiers in Pharmacology. (2025) 16, 10.3389/fphar.2025.1614587. PMC12343574 40808683

[20] Wang A. , Li H. , Xie Z. et al., Randomized, Placebo-Controlled, Multicenter Clinical Study on the Efficacy and Safety of Lidocaine Patches in Chinese Patients With Postherpetic Neuralgia, Dermatologic Therapy. (2023) 13, no. 7, 1477–1487, 10.1007/s13555-023-00938-8. PMC10307735 37268784

[21] Derry S. , Rice A. S. , Cole P. , Tan T. , and Moore R. A. , Topical Capsaicin (High Concentration) for Chronic Neuropathic Pain in Adults, Cochrane Database of Systematic Reviews. (2017) 1, no. 1. 10.1002/14651858.CD007393.pub4 PMC6464756 28085183

[22] Bundrant L. , Hunt T. L. , Banks P. et al., Results of Two Phase 1, Randomized, Double-Blind, Placebo-Controlled, Studies (Ascending Single-Dose and Multiple-Dose Studies) to Determine the Safety, Tolerability, and Pharmacokinetics of Orally Administered LX9211 in Healthy Participants, Clinical Therapeutics. (2021) 43, no. 6, 1029–1050, 10.1016/j.clinthera.2021.04.014. 34108079

[23] Bockbrader H. N. , Budhwani M. N. , and Wesche D. L. , Gabapentin to Pregabalin Therapy Transition: a Pharmacokinetic Simulation, American Journal of Therapeutics. (2013) 20, no. 1, 32–36, 10.1097/mjt.0b013e318250f80e. 23018586

[24] Cao X. , Shen Z. , Wang X. , Zhao J. , Liu W. , and Jiang G. , A Meta-Analysis of Randomized Controlled Trials Comparing the Efficacy and Safety of Pregabalin and Gabapentin in the Treatment of Postherpetic Neuralgia, Pain and Therapy. (2023) 12, no. 1, 1–18, 10.1007/s40122-022-00451-4. PMC9845451 36334235

[25] Pan T. , Ma J. , Li Y. et al., Rapid Onset of Pain Relief With Crisugabalin in Patients With Diabetic Peripheral Neuropathic Pain: Findings From a Multicenter, Randomized, Double-Blind, Controlled Study, Pain and Therapy. (2025) 14, no. 4, 1311–1329, 10.1007/s40122-025-00745-3. PMC12279680 40377855

[26] Gou X. , Liu Y. , Ye Q. et al., Crisugabalin, A Ligand for the α(2)δ Subunit of Voltage-Gated Calcium Channels, Exhibits no Obvious Abuse Potential in Rodents, Pharmacology, Biochemistry and Behavior. (2025) 252. 10.1016/j.pbb.2025.174015 40316144

[27] Van Oorschot D. , McGirr A. , Goulet P. et al., A Cross-Sectional Concept Elicitation Study to Understand the Impact of Herpes Zoster on Patients’ Health-Related Quality of Life, Infectious Disease and Therapy. (2022) 11, no. 1, 501–516, 10.1007/s40121-021-00581-w. PMC8847496 34994924

[28] Díez-Domingo J. , Curran D. , Cambronero M. D. R. , Garcia-Martinez J. A. , and Matthews S. , Economic Burden and Impact on Quality of Life of Herpes zoster in Spanish Adults Aged 50 Years or Older: A Prospective Cohort Study, Advances in Therapy. (2021) 38, no. 6, 3325–3341, 10.1007/s12325-021-01717-7. PMC8190024 34013498

[29] Ferini-Strambi L. , Neuropathic Pain and Sleep: A Review, Pain and Therapy. (2017) 6, no. Suppl 1, 19–23, 10.1007/s40122-017-0089-y. PMC5701896 29178036

[30] Dai W. , Kilpeläinen T. , Wen M. et al., Differential Effects of Pregabalin and Morphine on the Sleep-Wake Cycle and Circadian Rhythms in Mice With Neuropathic Pain, Anesthesiology. (2025) 143, no. 5, 1313–1339, 10.1097/aln.0000000000005715. PMC12513049 40802988

[31] Mercan A. , Uzun S. T. , Keles S. , Hacibeyoglu G. , Yilmaz R. , and Reisli R. , Immunological Mechanism of Postherpetic Neuralgia and Effect of Pregabalin Treatment on the Mechanism: A Prospective Single-Arm Observational Study, Korean Journal of Pain. (2021) 34, no. 4, 463–470, 10.3344/kjp.2021.34.4.463. PMC8494950 34593664

[32] Huang Y. , Xu C. , Zeng T. et al., Intravenous Patient-Controlled Analgesia Hydromorphone Combined With Pregabalin for the Treatment of Postherpetic Neuralgia: A Multicenter, Randomized Controlled Study, Korean Journal of Pain. (2021) 34, no. 2, 210–216, 10.3344/kjp.2021.34.2.210. PMC8019957 33785673

