# Supplementary material for: An Exploratory Retrospective Comparison of Crisugabalin and Pregabalin Among 12‐Week Treatment Completers With Postherpetic Neuralgia
Source: Pain Res Manag. 2026 Oct 12;2026:8540301. doi: 10.1155/prm/8540301 (PMC13655659; doi:10.1155/prm/8540301)
Supplement: Supplementary file 1 [file PRM-2026-8540301-s001.doc]

**Table Of Linear Mixed-Effects Model Supplemental Results**

**Supplementary Table 1
NRS Scoring Linear Mixed-Effects Model Fixed-Effects Estimates**

| **Parameter** | **Estimate** | **SE** | **df** | ***t*** | ***P*** | **95% CI** | |
| --- | --- | --- | --- | --- | --- | --- | --- |
|  |  |  |  |  |  | **LCL** | **UCL** |
| Intercept | 3.743 | 0.607 | 445.884 | 6.166 | <0.001 | 2.550 | 4.936 |
| **Intra-group Comparison** | | | | | | | |
| [Drug=1] | -1.820 | 0.284 | 112.824 | -6.413 | <0.001 | -2.382 | -1.258 |
| [Gender=1] | -0.091 | 0.129 | 437.246 | -0.706 | 0.481 | -0.344 | 0.162 |
| [Pain Location=1] | 0.668 | 0.204 | 437.246 | 3.276 | 0.001 | 0.267 | 1.069 |
| [Pain Location=2] | 0.793 | 0.179 | 437.246 | 4.418 | <0.001 | 0.440 | 1.145 |
| [Pain Location=3] | 0.362 | 0.217 | 437.246 | 1.669 | 0.096 | -0.064 | 0.788 |
| [Duration Of Pain Before Enrollment=1] | -0.418 | 0.314 | 437.246 | -1.332 | 0.184 | -1.036 | 0.199 |
| [Duration Of Pain Before Enrollment=2] | 0.163 | 0.426 | 437.246 | 0.382 | 0.702 | -0.674 | 0.999 |
| [Hypertension=0] | -0.435 | 0.145 | 437.246 | -3.001 | 0.003 | -0.719 | -0.150 |
| [Diabetes Mellitus=0] | -0.501 | 0.175 | 437.246 | -2.863 | 0.004 | -0.845 | -0.157 |
| Age | 0.001 | 0.005 | 437.246 | 0.195 | 0.846 | -0.009 | 0.011 |
| **Inter-group Comparison** | | | | | | | |
| [Time=1] | 2.947 | 0.252 | 212.423 | 11.712 | <0.001 | 2.451 | 3.443 |
| [Time=2] | 1.807 | 0.258 | 214.719 | 6.995 | <0.001 | 1.298 | 2.316 |
| [Time=3] | 0.825 | 0.267 | 217.266 | 3.083 | 0.002 | 0.297 | 1.352 |
| **Interaction Effect** | | | | | | | |
| [Time=1] * [Drug=1] | 1.410 | 0.357 | 212.423 | 3.944 | <0.001 | 0.705 | 2.114 |
| [Time=2] * [Drug=1] | 0.425 | 0.367 | 214.719 | 1.159 | 0.248 | -0.298 | 1.148 |
| [Time=3] * [Drug=1] | 0.122 | 0.380 | 217.266 | 0.321 | 0.749 | -0.627 | 0.871 |

*Coding and reference categories*: Drug: 1=CGB group, 2=PGB group, reference=2; Gender: 1=Man, 2=Woman, reference=2; Pain Location: 1=Abdomen, 2=Chest and back, 3=Limbs, 4=Others, reference=4; Duration Of Pain Before Enrollment: 1=3 < t ≤ 6, 2=6 < t ≤ 12, 3=12 ≤ t, reference=3; Hypertension: 0=No, 1=Yes, reference=1; Diabetes Mellitus: 0=No, 1=Yes, reference=1

**Supplementary Table 2
Intra-group And Inter-group NRS Score Changes And Post-hoc Tests**

| **Comparative Grouping** | | **MD** | **SE** | **df** | ***P*** | **95% CI** | |
| --- | --- | --- | --- | --- | --- | --- | --- |
| **Inter-group Comparison** | | | | | | | |
| Drug | Drug |  |  |  |  | LCL | UCL |
| CGB Group | PGB Group | -1.331 | 0.130 | 441.053 | <0.001 | -1.587 | -1.074 |
| **Intra-group Comparison** | | | | | | | |
| Time | Time |  |  |  |  | LCL | UCL |
| T0 | T1 | 1.633 | 0.162 | 219.349 | <0.001 | 1.203 | 2.063 |
|  | T4 | 2.767 | 0.169 | 218.790 | <0.001 | 2.317 | 3.216 |
|  | T12 | 3.652 | 0.179 | 212.423 | <0.001 | 3.176 | 4.128 |
| T1 | T0 | -1.633 | 0.162 | 219.349 | <0.001 | -2.063 | -1.203 |
|  | T4 | 1.134 | 0.174 | 222.514 | <0.001 | 0.671 | 1.597 |
|  | T12 | 2.020 | 0.183 | 214.719 | <0.001 | 1.531 | 2.508 |
| T4 | T0 | -2.767 | 0.169 | 218.790 | <0.001 | -3.216 | -2.317 |
|  | T1 | -1.134 | 0.174 | 222.514 | <0.001 | -1.597 | -0.671 |
|  | T12 | 0.885 | 0.190 | 217.266 | <0.001 | 0.380 | 1.391 |
| T12 | T0 | -3.652 | 0.179 | 212.423 | <0.001 | -4.128 | -3.176 |
|  | T1 | -2.020 | 0.183 | 214.719 | <0.001 | -2.508 | -1.531 |
|  | T4 | -0.885 | 0.190 | 217.266 | <0.001 | -1.391 | -0.380 |
| **Medication-by-Time Estimated Marginal Means** | | | | | | | |
| Drug | **Time** | **Average** |  |  |  | LCL | UCL |
| CGB Group | T0 | 6.208 | 0.207 | 206.692 | - | 5.799 | 6.617 |
|  | T1 | 4.083 | 0.216 | 209.952 | - | 3.658 | 4.508 |
|  | T4 | 2.797 | 0.227 | 204.211 | - | 2.350 | 3.244 |
|  | T12 | 1.851 | 0.242 | 191.976 | - | 1.374 | 2.327 |
| PGB Group | T0 | 6.618 | 0.223 | 238.420 | - | 6.178 | 7.058 |
|  | T1 | 5.477 | 0.231 | 242.464 | - | 5.023 | 5.932 |
|  | T4 | 4.495 | 0.241 | 236.874 | - | 4.020 | 4.970 |
|  | T12 | 3.670 | 0.255 | 223.433 | - | 3.168 | 4.172 |

**Supplementary Table 3
PSQI Scoring Linear Mixed-Effects Model Fixed-Effects Estimates**

| **Parameter** | **Estimate** | **SE** | **df** | ***t*** | ***P*** | **95% CI** | |
| --- | --- | --- | --- | --- | --- | --- | --- |
|  |  |  |  |  |  | **LCL** | **UCL** |
| Intercept | 11.705 | 1.459 | 447.812 | 8.024 | <0.001 | 8.838 | 14.572 |
| **Intra-group Comparison** | | | | | | | |
| [Drug=1] | -3.816 | 0.663 | 114.167 | -5.757 | <0.001 | -5.130 | -2.503 |
| [Gender=1] | -0.995 | 0.310 | 444.410 | -3.206 | 0.001 | -1.605 | -0.385 |
| [Pain Location=1] | 0.780 | 0.492 | 444.410 | 1.585 | 0.114 | -0.187 | 1.746 |
| [Pain Location=2] | 1.156 | 0.433 | 444.410 | 2.672 | 0.008 | 0.306 | 2.006 |
| [Pain Location=3] | 0.370 | 0.523 | 444.410 | 0.708 | 0.479 | -0.657 | 1.398 |
| [Duration Of Pain Before Enrollment=1] | -1.722 | 0.757 | 444.410 | -2.274 | 0.023 | -3.210 | -0.234 |
| [Duration Of Pain Before Enrollment=2] | -1.476 | 1.026 | 444.410 | -1.439 | 0.151 | -3.492 | 0.540 |
| [Hypertension=0] | -0.707 | 0.349 | 444.410 | -2.023 | 0.044 | -1.393 | -0.020 |
| [Diabetes Mellitus=0] | -0.913 | 0.422 | 444.410 | -2.162 | 0.031 | -1.742 | -0.083 |
| Age | 0.010 | 0.013 | 444.410 | 0.809 | 0.419 | -0.015 | 0.035 |
| **Inter-group Comparison** | | | | | | | |
| [Time=1] | 5.035 | 0.604 | 218.752 | 8.336 | <0.001 | 3.845 | 6.226 |
| [Time=2] | 3.386 | 0.612 | 218.701 | 5.537 | <0.001 | 2.181 | 4.591 |
| [Time=3] | 1.456 | 0.627 | 218.709 | 2.321 | 0.021 | 0.219 | 2.693 |
| Interaction Effect |  |  |  |  |  |  |  |
| [Time=1] * [Drug=1] | 3.215 | 0.858 | 218.752 | 3.747 | <0.001 | 1.524 | 4.906 |
| [Time=2] * [Drug=1] | 1.043 | 0.869 | 218.701 | 1.200 | 0.231 | -0.669 | 2.755 |
| [Time=3] * [Drug=1] | 0.151 | 0.891 | 218.709 | 0.169 | 0.866 | -1.606 | 1.908 |

**Supplementary Table 4
 Intra-group And Inter-group PSQI Changes And Post-hoc Tests**

| **Comparative Grouping** | | **MD** | **SE** | **df** | ***P*** | **95% CI** | |
| --- | --- | --- | --- | --- | --- | --- | --- |
| **Inter-group Comparison** | | | | | | | |
| Drug | Drug |  |  |  |  | LCL | UCL |
| CGB Group | PGB Group | -2.714 | 0.312 | 446.078 | <0.001 | -3.328 | -2.101 |
| **Intra-group Comparison** | | | | | | | |
| Time | Time |  |  |  |  | LCL | UCL |
| T0 | T1 | 2.735 | 0.397 | 220.554 | <0.001 | 1.679 | 3.791 |
|  | T4 | 5.111 | 0.409 | 222.660 | <0.001 | 4.022 | 6.200 |
|  | T12 | 6.643 | 0.429 | 218.752 | <0.001 | 5.500 | 7.785 |
| T1 | T0 | -2.735 | 0.397 | 220.554 | <0.001 | -3.791 | -1.679 |
|  | T4 | 2.376 | 0.415 | 223.611 | <0.001 | 1.272 | 3.479 |
|  | T12 | 3.907 | 0.434 | 218.701 | <0.001 | 2.751 | 5.064 |
| T4 | T0 | -5.111 | 0.409 | 222.660 | <0.001 | -6.200 | -4.022 |
|  | T1 | -2.376 | 0.415 | 223.611 | <0.001 | -3.479 | -1.272 |
|  | T12 | 1.532 | 0.446 | 218.709 | 0.004 | 0.345 | 2.718 |
| T12 | T0 | -6.643 | 0.429 | 218.752 | <0.001 | -7.785 | -5.500 |
|  | T1 | -3.907 | 0.434 | 218.701 | <0.001 | -5.064 | -2.751 |
|  | T4 | -1.532 | 0.446 | 218.709 | 0.004 | -2.718 | -0.345 |
| **Medication-by-Time Estimated Marginal Means** | | | | | | | |
| Drug | **Time** | **Average** |  |  |  | LCL | UCL |
| CGB Group | T0 | 15.046 | 0.511 | 206.318 | - | 14.038 | 16.053 |
|  | T1 | 11.224 | 0.520 | 211.131 | - | 10.199 | 12.250 |
|  | T4 | 8.403 | 0.539 | 206.273 | - | 7.340 | 9.466 |
|  | T12 | 6.796 | 0.570 | 196.730 | - | 5.672 | 7.919 |
| PGB Group | T0 | 15.647 | 0.549 | 238.786 | - | 14.567 | 16.728 |
|  | T1 | 13.998 | 0.557 | 243.882 | - | 12.901 | 15.095 |
|  | T4 | 12.068 | 0.574 | 239.215 | - | 10.937 | 13.199 |
|  | T12 | 10.612 | 0.603 | 228.871 | - | 9.425 | 11.799 |

**Supplementary Table 5
Fixed-Effects Estimates Of Linear Mixed Effects Model For SF-36 Physical Component Assessment**

| **Parameter** | **Estimate** | **SE** | **df** | ***t*** | ***P*** | **95% CI** | |
| --- | --- | --- | --- | --- | --- | --- | --- |
|  |  |  |  |  |  | **LCL** | **UCL** |
| Intercept | 43.758 | 2.278 | 447.718 | 19.211 | <0.001 | 39.282 | 48.235 |
| **Intra-group Comparison** | | | | | | | |
| [Drug=1] | 3.565 | 1.047 | 115.234 | 3.406 | 0.001 | 1.492 | 5.638 |
| [Gender=1] | 2.836 | 0.484 | 443.011 | 5.860 | <0.001 | 1.885 | 3.787 |
| [Pain Location=1] | 1.062 | 0.767 | 443.011 | 1.384 | 0.167 | -0.445 | 2.568 |
| [Pain Location=2] | 1.033 | 0.675 | 443.011 | 1.531 | 0.127 | -0.293 | 2.358 |
| [Pain Location=3] | 0.483 | 0.816 | 443.011 | 0.592 | 0.554 | -1.120 | 2.086 |
| [Duration Of Pain Before Enrollment=1] | 4.196 | 1.181 | 443.011 | 3.553 | <0.001 | 1.875 | 6.517 |
| [Duration Of Pain Before Enrollment=2] | 4.133 | 1.600 | 443.011 | 2.583 | 0.010 | 0.989 | 7.278 |
| [Hypertension=0] | 0.119 | 0.545 | 443.011 | 0.219 | 0.827 | -0.951 | 1.190 |
| [Diabetes Mellitus=0] | 0.400 | 0.658 | 443.011 | 0.608 | 0.543 | -0.893 | 1.694 |
| Age | -0.076 | 0.020 | 443.011 | -3.848 | <0.001 | -0.115 | -0.037 |
| **Inter-group Comparison** | | | | | | | |
| [Time=1] | -5.140 | 0.985 | 223.518 | -5.217 | <0.001 | -7.082 | -3.199 |
| [Time=2] | -3.333 | 0.944 | 216.931 | -3.530 | 0.001 | -5.194 | -1.472 |
| [Time=3] | -1.649 | 0.962 | 218.696 | -1.714 | 0.088 | -3.545 | 0.247 |
| **Interaction Effect** | | | | | | | |
| [Time=1] * [Drug=1] | -2.181 | 1.400 | 223.518 | -1.558 | 0.121 | -4.939 | 0.577 |
| [Time=2] * [Drug=1] | -0.810 | 1.341 | 216.931 | -0.604 | 0.547 | -3.453 | 1.834 |
| [Time=3] * [Drug=1] | -0.262 | 1.367 | 218.696 | -0.191 | 0.848 | -2.955 | 2.432 |

**Supplementary Table 6
 Intra-group And Inter-group SF-36 Physical Changes And Post-hoc Tests**

| **Comparative Grouping** | | **MD** | **SE** | **df** | ***P*** | **95% CI** | |
| --- | --- | --- | --- | --- | --- | --- | --- |
| **Inter-group Comparison** | | | | | | | |
| Drug | Drug |  |  |  |  | LCL | UCL |
| CGB Group | PGB Group | 2.752 | 0.488 | 444.910 | <0.001 | 1.793 | 3.711 |
| **Intra-group Comparison** | | | | | | | |
| Time | Time |  |  |  |  | LCL | UCL |
| T0 | T1 | -2.493 | 0.634 | 221.610 | 0.001 | -4.181 | -0.804 |
|  | T4 | -4.451 | 0.648 | 224.829 | <0.001 | -6.175 | -2.727 |
|  | T12 | -6.231 | 0.700 | 223.518 | <0.001 | -8.094 | -4.368 |
| T1 | T0 | 2.493 | 0.634 | 221.610 | 0.001 | 0.804 | 4.181 |
|  | T4 | -1.958 | 0.616 | 225.247 | 0.010 | -3.598 | -0.319 |
|  | T12 | -3.738 | 0.671 | 216.931 | <0.001 | -5.524 | -1.953 |
| T4 | T0 | 4.451 | 0.648 | 224.829 | <0.001 | 2.727 | 6.175 |
|  | T1 | 1.958 | 0.616 | 225.247 | 0.010 | 0.319 | 3.598 |
|  | T12 | -1.780 | 0.683 | 218.696 | 0.059 | -3.599 | 0.039 |
| T12 | T0 | 6.231 | 0.700 | 223.518 | <0.001 | 4.368 | 8.094 |
|  | T1 | 3.738 | 0.671 | 216.931 | <0.001 | 1.953 | 5.524 |
|  | T4 | 1.780 | 0.683 | 218.696 | 0.059 | -0.039 | 3.599 |
| **Medication-by-Time Estimated Marginal Means** | | | | | | | |
| Drug | **Time** | **Average** |  |  |  | LCL | UCL |
| CGB Group | T0 | 39.883 | 0.841 | 206.019 | - | 38.225 | 41.541 |
|  | T1 | 43.062 | 0.791 | 213.833 | - | 41.502 | 44.622 |
|  | T4 | 45.294 | 0.813 | 211.285 | - | 43.691 | 46.897 |
|  | T12 | 47.205 | 0.896 | 196.797 | - | 45.438 | 48.972 |
| PGB Group | T0 | 38.500 | 0.896 | 238.791 | - | 36.735 | 40.264 |
|  | T1 | 40.307 | 0.850 | 246.143 | - | 38.632 | 41.981 |
|  | T4 | 41.991 | 0.870 | 244.022 | - | 40.277 | 43.705 |
|  | T12 | 43.640 | 0.947 | 228.660 | - | 41.775 | 45.505 |

**Supplementary Table 7
Fixed-Effects Estimates Of Linear Mixed Effects Model For SF-36 Mental Component Summary Assessment**

| **Parameter** | **Estimate** | **SE** | **df** | ***t*** | ***P*** | **95% CI** | |
| --- | --- | --- | --- | --- | --- | --- | --- |
|  |  |  |  |  |  | **LCL** | **UCL** |
| Intercept | 43.087 | 2.920 | 445.734 | 14.757 | <0.001 | 37.348 | 48.825 |
| **Intra-group Comparison** | | | | | | | |
| [Drug=1] | 8.403 | 1.353 | 113.198 | 6.210 | <0.001 | 5.722 | 11.084 |
| [Gender=1] | 1.971 | 0.620 | 438.992 | 3.181 | 0.002 | 0.753 | 3.189 |
| [Pain Location=1] | -0.809 | 0.982 | 438.992 | -0.824 | 0.410 | -2.738 | 1.121 |
| [Pain Location=2] | -1.242 | 0.864 | 438.992 | -1.438 | 0.151 | -2.940 | 0.456 |
| [Pain Location=3] | -0.820 | 1.044 | 438.992 | -0.785 | 0.433 | -2.873 | 1.232 |
| [Duration Of Pain Before Enrollment=1] | 3.653 | 1.512 | 438.992 | 2.416 | 0.016 | 0.681 | 6.625 |
| [Duration Of Pain Before Enrollment=2] | 2.909 | 2.049 | 438.992 | 1.420 | 0.156 | -1.117 | 6.936 |
| [Hypertension=0] | 1.445 | 0.697 | 438.992 | 2.072 | 0.039 | 0.074 | 2.816 |
| [Diabetes Mellitus=0] | 1.334 | 0.843 | 438.992 | 1.583 | 0.114 | -0.322 | 2.991 |
| Age | -0.070 | 0.025 | 438.992 | -2.761 | 0.006 | -0.120 | -0.020 |
| **Inter-group Comparison** | | | | | | | |
| [Time=1] | -7.070 | 1.264 | 222.571 | -5.594 | <0.001 | -9.561 | -4.579 |
| [Time=2] | -5.088 | 1.201 | 211.757 | -4.236 | <0.001 | -7.455 | -2.720 |
| [Time=3] | -2.123 | 1.260 | 214.754 | -1.685 | 0.093 | -4.606 | 0.360 |
| **Interaction Effect** | | | | | | | |
| [Time=1] * [Drug=1] | -6.126 | 1.795 | 222.571 | -3.412 | 0.001 | -9.664 | -2.588 |
| [Time=2] * [Drug=1] | -2.019 | 1.706 | 211.757 | -1.184 | 0.238 | -5.382 | 1.343 |
| [Time=3] * [Drug=1] | -1.038 | 1.789 | 214.754 | -0.580 | 0.563 | -4.565 | 2.489 |

**Supplementary Table 8
 Intra-group And Inter-group SF-36 Mental Changes And Post-hoc Testing**

| **Comparative Grouping** | | **MD** | **SE** | **df** | ***P*** | **95% CI** | |
| --- | --- | --- | --- | --- | --- | --- | --- |
| **Inter-group Comparison** | | | | | | | |
| Drug | Drug |  |  |  |  | LCL | UCL |
| CGB Group | PGB Group | 6.107 | 0.627 | 443.012 | <0.001 | 4.875 | 7.339 |
| **Intra-group Comparison** | | | | | | | |
| Time | Time |  |  |  |  | LCL | UCL |
| T0 | T1 | -4.036 | 0.797 | 214.726 | <0.001 | -6.158 | -1.914 |
|  | T4 | -7.492 | 0.841 | 224.450 | <0.001 | -9.731 | -5.252 |
|  | T12 | -10.133 | 0.898 | 222.571 | <0.001 | -12.523 | -7.744 |
| T1 | T0 | 4.036 | 0.797 | 214.726 | <0.001 | 1.914 | 6.158 |
|  | T4 | -3.456 | 0.793 | 222.675 | <0.001 | -5.568 | -1.343 |
|  | T12 | -6.097 | 0.853 | 211.757 | <0.001 | -8.369 | -3.826 |
| T4 | T0 | 7.492 | 0.841 | 224.450 | <0.001 | 5.252 | 9.731 |
|  | T1 | 3.456 | 0.793 | 222.675 | <0.001 | 1.343 | 5.568 |
|  | T12 | -2.642 | 0.895 | 214.754 | 0.021 | -5.024 | -0.259 |
| T12 | T0 | 10.133 | 0.898 | 222.571 | <0.001 | 7.744 | 12.523 |
|  | T1 | 6.097 | 0.853 | 211.757 | <0.001 | 3.826 | 8.369 |
|  | T4 | 2.642 | 0.895 | 214.754 | 0.021 | 0.259 | 5.024 |
| **Medication-by-Time Estimated Marginal Means** | | | | | | | |
| Drug | **Time** | **Average** |  |  |  | LCL | UCL |
| CGB Group | T0 | 37.346 | 1.071 | 202.870 | - | 35.233 | 39.458 |
|  | T1 | 43.435 | 0.995 | 213.311 | - | 41.474 | 45.396 |
|  | T4 | 47.381 | 1.066 | 207.316 | - | 45.279 | 49.483 |
|  | T12 | 50.542 | 1.155 | 193.213 | - | 48.264 | 52.820 |
| PGB Group | T0 | 35.069 | 1.142 | 235.388 | - | 32.820 | 37.318 |
|  | T1 | 37.051 | 1.071 | 245.009 | - | 34.941 | 39.162 |
|  | T4 | 40.016 | 1.137 | 240.133 | - | 37.776 | 42.256 |
|  | T12 | 42.139 | 1.219 | 224.799 | - | 39.736 | 44.542 |

**Supplementary Table 9**

**Mauchly's Sphericity Test In RM-ANOVA**

| **Measure** | **W** | **X2** | **P** |
| --- | --- | --- | --- |
| NRS | 0.451 | 80.119 | <0.001 |
| PSQI | 0.320 | 114.862 | <0.001 |
| HAMD-17 | 0.178 | 173.826 | <0.001 |
| SF-36 Physical | 0.399 | 92.504 | <0.001 |
| SF-36 Mental | 0.169 | 179.113 | <0.001 |
